# Comparison of Vitamin D Levels and Related Factors in Pregnant Women and Neonates Exposed to Second-Hand Smoke

**DOI:** 10.7759/cureus.28287

**Published:** 2022-08-23

**Authors:** Süleyman Yıldız, Ömer Tammo

**Affiliations:** 1 Pediatrics and Child Health, Mardin Derik State Hospital, Mardin, TUR; 2 Obstetrics and Gynaecology, Mardin State Hospital, Mardin, TUR

**Keywords:** cigarette, pregnancy, neonates, vitamin d, second-hand smoking

## Abstract

Introduction: Exposure to second-hand smoke, a significant public health issue today, may lead to various health problems, especially in pregnant women and their infants. Low vitamin D levels during pregnancy may lead to preeclampsia and gestational diabetes in the mother, while it may cause low birth weight and respiratory problems in the infant.

Method: The study group consisted of 42 mothers, who smoked regularly, and their infants and 45 mothers (passive smokers), who were regularly exposed to second-hand smoke in their home environment, although they did not smoke, and their infants. Meanwhile, the control group consisted of 46 healthy mothers, who did not smoke and were not exposed to second-hand smoke at home, and their infants with similar gestational age and birth weight. Blood samples were taken as two different samples, from the mother and the baby, and 25-hydroxyvitamin D (25(OH)D) and related blood parameters were studied and compared statistically.

Results: 25(OH)D, calcium, and magnesium levels of mothers who smoked were significantly lower than those who were exposed to second-hand smoke and those who did not. Moreover, the vitamin D levels of mothers and babies exposed to second-hand smoke in the non-smoker group were significantly lower than mothers and babies who were not exposed to second-hand smoke. In the babies of these three groups, a significant decrease was observed only in vitamin D levels.

Conclusion: The present study shows that pregnant women and their infants exposed to second-hand smoke have lower vitamin D levels. Hence, more emphasis should be put on vitamin D monitoring and supplementation to prevent severe health problems in pregnant women and their infants exposed to tobacco smoke. Further studies are needed to assess the associated risks for maternal and fetal health as well as possible long-term implications for the infant.

## Introduction

During pregnancy, various complications, such as preeclampsia, small for gestational age, gestational diabetes, and recurrent miscarriages occur due to vitamin D deficiency [1]. Nutrition, obesity, physical activity, and active or passive exposure to tobacco smoke impact the vitamin D metabolite, 25-hydroxyvitamin D (25(OH)D), and thus the mother's and baby's bone metabolism [2]. In particular, it has been suggested that the most notable reason why smoking causes low bone density and bone fractures is the suppression of vitamin D synthesis in the body [3].

Today, smoking continues to be an important public health problem. In Turkey, roughly 25,000 people die every year due to second-hand smoke even though they do not smoke [4]. The most important groups affected by second-hand smoke are infants and pregnant women. Exposure to second-hand smoke during the prenatal period may adversely impact the health of both groups as much as active smoking [5].

In the province where we have conducted the present study (Mardin, Turkey), the prevalence of vitamin D deficiency and second-hand smoking during pregnancy is relatively high [4,6]. In this study, we aimed to evaluate vitamin D levels and some related biochemical parameters in pregnant women who smoked regularly during pregnancy, as well as in mothers and newborns who did not actively smoke but were exposed to second-hand smoke.

## Materials and methods

The present study was conducted prospectively between July 2021 and October 2021. The study group consisted of 42 mothers, who smoked regularly during pregnancy (at least one cigarette a day), and their infants and 45 mothers (passive smokers), who were regularly exposed to second-hand smoke in their home environment, although they did not smoke, and their infants. Meanwhile, the control group consisted of 46 healthy mothers, who did not smoke and were not exposed to second-hand smoke at home, and their infants with similar gestational age and birth weight. Smoking status and exposure to tobacco smoke were through administering a questionnaire prepared by the researchers. Pregnant women who were at term of delivery (37-41 weeks) and who did not experience any complications during their follow-up and delivery were included in the present study. Pregnant women with any chronic disease or psychological disorder or use of toxic substances other than cigarettes (such as alcohol) and newborns with acute or chronic illness were excluded from this study.

Written informed consent was obtained from the mothers included in this study, ethical approval was obtained from the local ethics committee (study ethics committee number: E-91872217-929), and the Declaration of Helsinki ethical principles were followed.

Collection and measurement of blood samples

Blood samples were taken as two different samples, from the mother and the baby. To determine 25(OH)D levels and related blood parameters (calcium, magnesium, and phosphorus) levels, 5 cc blood samples were taken from the mother's upper extremity vein before delivery and from the umbilical vein in newborns just after delivery. After the samples were centrifuged at 4000 rpm for six minutes, they were stored at -20°C until the analysis day. Serum 25(OH)D concentrations were measured by a chemiluminescence assay using the LIAISON instrument with the DiaSorin kit (DiaSorin Inc., Stillwater, MN).

In our study, vitamin D deficiency was considered to be 25(OH)D level < 20 ng/mL (50 nmol/liter), while vitamin D insufficiency was considered to be 25(OH)D level between 21 and 29 ng/ml (525-725 nmol/liter) [6].

Statistical method

SPSS 15.0 for Windows software (SPSS Inc., Chicago, IL) was used for statistical analysis. Descriptive statistics were expressed as numbers and percentages for categorical variables, and they were expressed as mean, standard deviation, minimum, maximum, and median for numerical variables. The rates in the groups were compared using the chi-square test. Independent samples t-test of numerical variables were made using the Kruskal-Wallis test since the normal distribution condition was not met. Subgroup analyses were conducted using the Mann-Whitney U test and interpreted with Bonferroni correction. The correlations between numerical variables were analyzed by Spearman correlation analysis since the parametric test condition was not met. Determining factors were done by linear regression analysis. The results were considered significant at p ˂ 0.05.

## Results

A significant difference was determined in the smoker groups in maternal characteristics, such as age (p = 0.003), gestational week (p = 0.018), systolic blood pressure (p < 0.001), diastolic blood pressure (p < 0.001), and proteinuria levels (p < 0.001). The gestational week of the mothers who smoked was significantly lower than the mothers who were exposed to second-hand smoke and those who did not smoke, whereas systolic and diastolic blood pressures and proteinuria levels were significantly higher (week of gestation: p = 0.017 for both, other comparisons: p < 0.001) (Table 1).

**Table 1:** Comparison of demographic and clinical characteristics between groups * Kruskal-Wallis test. APGAR: appearance, pulse, grimace, activity, and respiration.

	Smoker		Exposed to second-hand smoke		Non-smoker		
	Mean ± SD	Median (Min-Max)	Mean ± SD	Median (Min-Max)	Mean ± SD	Median (Min-Max)	P*
Mothers' characteristics							
Age (years)	28.6 ± 5.7	27 (21-42)	29.0 ± 9.2	26 (20-52)	34.4 ± 10.2	33 (19-59)	0.003
Gestational week	38.2 ± 1.0	38 (36-41)	38.6 ± 0.7	39 (37-39)	38.6 ± 0.7	39 (37-39)	0.018
Systolic (mmHg)	141.4 ± 13.1	135 (120-170)	117.7 ± 10.1	120 (95-153)	113.3 ± 14.1	115 (90-153)	<0.001
Diastolic (mmHg)	88.2 ± 10.8	90 (60-115)	73.2 ± 8.9	75 (60-95)	72.4 ± 8.8	70 (59-105)	<0.001
Proteinuria (mg/dL)	1.27 ± 0.84	1 (0-3)	0.42 ± 0.75	0 (0-3)	0.38 ± 0.61	0 (0-2)	<0.001
Infants' characteristics							
Week	38.2 ± 1.3	38 (37-40)	38.5 ± 0.9	39 (37-41)	38.4 ± 1.0	39 (37-41)	0.545
Weight (gram)	3159.8 ± 386.0	3085 (2500-3940)	3547.1 ± 308.9	3650 (2740-3910)	3588.0 ± 261.7	3640 (2880-4100)	<0.001
Height (cm)	46.4 ± 4.7	48 (37-52)	49.1 ± 2.0	49 (40-52)	49.4 ± 1.9	50 (41-53)	0.001
Head circumference (cm)	36.4 ± 1.9	37 (32-39)	36.3 ± 1.3	36 (34-39)	36.3 ± 1.2	36 (35-39)	0.572
1-minute APGAR	6.8 ± 0.9	7 (5-8)	7.4 ± 0.9	7 (5-9)	7.2 ± 1.0	7 (5-9)	0.007
5-minute APGAR	9.2 ± 0.7	9 (7-10)	9.4 ± 0.5	9 (8-10)	9.4 ± 0.5	9 (9-10)	0.173

A significant difference was found in the smoker groups regarding weight (p < 0.001), height (p = 0.001), and one-minute APGAR (appearance, pulse, grimace, activity, and respiration) scores (p = 0.007). Weight (p < 0.001), height (p < 0.001), and one-minute APGAR scores (p = 0.003) were significantly lower in infants of mothers who were exposed to second-hand smoke than in infants of non-smoking mothers (Tables 1, 2).

**Table 2:** Subgroup analyses * Mann-Whitney U test Bonferroni correction (p < 0.017). APGAR: appearance, pulse, grimace, activity, and respiration.

	Smoker vs. exposed to second-hand smoke	Smoker vs. non-smoker	Exposed to second-hand smoke, non-smoker
Mother	p*	p*	p*
Age	0.419	0.005	0.002
Gestational week	0.017	0.017	1.000
Systolic blood pressure	<0.001	<0.001	0.156
Diastolic blood pressure	<0.001	<0.001	0.650
Proteinuria	<0.001	<0.001	0.976
Infants' characteristics			
Weight	<0.001	<0.001	0.618
Height	0.003	0.001	0.540
1-minute APGAR	0.002	0.030	0.401

A significant difference was found between the smoking groups regarding the levels of calcium, magnesium, and vitamin D in the laboratory characteristics of the mothers (p < 0.001 for all). Calcium (p < 0.001), magnesium (p < 0.001), and 25(OH)D (p < 0.001) levels of mothers who smoked regularly were significantly lower than those who were exposed to second-hand smoke and those who did not smoke (Tables 3, 4).

**Table 3:** 25(OH)D, calcium, magnesium, and phosphorus levels in the groups * Kruskal-Wallis test. 25(OH)D: 25-hydroxyvitamin D.

	Smoker		Exposed to second-hand smoke		Non-smoker		
Mothers' characteristics	Mean ± SD	Median (Min-Max)	Mean ± SD	Median (Min-Max)	Mean ± SD	Median (Min-Max)	P*
Calcium (mg/dl)	8.64 ± 0.50	8.6 (7.9-10)	9.11 ± 0.51	9.1 (8-10.5)	8.91 ± 0.55	9 (7-9.8)	<0.001
Magnesium (mg/dl)	2.47 ± 0.5	2.4 (1.8-4.7)	2.17 ± 0.20	2.2 (1.6-2.8)	2.17 ± 0.20	2.2 (1.8-3.1)	<0.001
25(OH)D (ng/ml)	21.5 ± 16.3	19 (5-89)	38.3 ± 18.5	35 (10-101)	56.5 ± 17.4	55 (20-97)	<0.001
Phosphorus (mg/dl)	3.29 ± 0.59	3.3 (2.2-4.7)	3.40 ± 0.63	3.6 (2-4.1)	3.12 ± 0.67	3.1 (1.9-4.2)	0.092
Infants' characteristics							
25(OH)D (ng/ml)	23.4 ± 13.7	20 (10-60)	31.4 ± 13.9	31 (21-77)	34.7 ± 13.7	56 (15-91)	0.001
Calcium (mg/dl)	9.1 ± 0.7	8.95 (7.8-11)	9.1 ± 0.5	9 (8.2-14)	9.2 ± 0.5	9 (8-12)	0.821
Magnesium (mg/dl)	2.2 ± 0.3	2.2 (1.2-4)	2.1 ± 0.2	2 (1.5-2.8)	2.2 ± 0.3	2.1 (1.7-3)	0.063
Phosphorus (mg/dl)	3.4 ± 0.7	3.65 (2-4.1)	3.3 ± 0.7	3.6 (2-4.3)	3.3 ± 0.6	3.5 (2-4.7)	0.561
Gender, M/F n (%)	20 (47.6%)/22 (52.4%)		23 (51.1%)/22 (48.9%)		17 (37.8%)/28 (62.2%)		0.421

**Table 4:** Subgroup analyses * Mann-Whitney U test Bonferroni correction (p < 0.017). 25(OH)D: 25-hydroxyvitamin D.

	Smoker vs. exposed to second-hand smoke	Smoker vs. non-smoker	Exposed to second-hand smoke, non-smoker
Mothers' characteristics	p*	p*	p*
Calcium (mg/dl)	<0.001	0.004	0.134
Magnesium (mg/dl)	0.004	0.004	1.000
25(OH)D (ng/ml)	<0.001	<0.001	<0.001
Phosphorus (mg/dl)	0.269	0.224	0.035
Infants' characteristics			
25(OH)D (ng/ml)	<0.001	<0.001	<0.001

A significant difference was found between infants of these groups only regarding vitamin D levels (p < 0.001 for all). Calcium and magnesium levels of infants of smoking mothers were significantly lower than those exposed to second-hand smoke and non-smokers (p = 0.001 for both) (Tables 3, 4).

Maternal calcium levels were negatively correlated with diastolic blood pressure (p < 0.001), maternal magnesium levels were positively correlated with systolic blood pressure (p = 0.019) and proteinuria (p = 0.043) levels, while maternal vitamin D levels were negatively correlated with systolic blood pressure (p < 0.001), diastolic blood pressure (p < 0.001), and proteinuria (p < 0.001) levels (Table 5).

**Table 5:** Comparison of calcium, magnesium, 25(OH)D, and phosphorus levels with clinical characteristics 25(OH)D: 25-hydroxyvitamin D; APGAR: appearance, pulse, grimace, activity, and respiration.

	Calcium		Magnesium		25(OH)D		Phosphorus	
Mothers' characteristics	r	p	r	p	r	p	r	p
Age	-0.046	0.594	-0.089	0.304	0.108	0.211	-0.033	0.708
Gestational week	0.057	0.513	0.03	0.729	0.128	0.138	-0.006	0.949
Systolic blood pressure	-0.122	0.158	0.201	0.019	-0.459	<0.001	-0.001	0.991
Diastolic blood pressure	-0.334	<0.001	0.143	0.099	-0.338	<0.001	-0.11	0.203
Proteinuria	-0.161	0.062	0.174	0.043	-0.316	<0.001	-0.004	0.968
Infants' characteristics								
Week	0.192	0.027	0.086	0.326	0.151	0.084	-0.022	0.805
Weight	0.050	0.570	0.007	0.938	0.242	0.005	0.098	0.263
Height	-0.114	0.192	0.116	0.186	-0.106	0.225	-0.150	0.086
Head circumference	0.074	0.401	-0.094	0.283	-0.117	0.181	-0.025	0.780
1-minute APGAR	0.066	0.453	-0.104	0.237	-0.105	0.232	-0.072	0.412
5-minute APGAR	0.043	0.625	0.056	0.523	-0.081	0.356	0.022	0.802

The infants' calcium level was positively correlated with the gestational week, and the 25(OH)D level was significantly correlated with weight (p = 0.027 and p = 0.005) (Table 5).

When the effects of smoking were corrected with maternal and infant characteristics and examined as the factor determining vitamin D level, it was a statistically significant factor (mother: p < 0.001; infant: p = 0.036) (Table 6).

**Table 6:** Multivariate linear regression analysis of factors determining 25(OH)D level 25(OH)D: 25-hydroxyvitamin D; APGAR: appearance, pulse, grimace, activity, and respiration.

Mothers' characteristics	B	Beta	P
Constant	-17.176		
Age	-0.010	-0.004	0.954
Gestational week	0.447	0.016	0.817
Systolic blood pressure	-0.044	-0.034	0.714
Diastolic blood pressure	0.123	0.065	0.445
Proteinuria	-0.387	-0.015	0.847
Group	17.623	0.644	<0.001
Dependent variable: 25(OH)D			
Infants' characteristics	B	Beta	P
Constant	58.725		
Week	0.143	0.011	0.911
Weight	0.005	0.135	0.199
Height	-0.462	-0.110	0.270
Head circumference	-0.913	-0.094	0.289
1-minute APGAR	-1.275	-0.085	0.496
5-minute APGAR	-0.997	-0.041	0.737
Calcium	0.668	0.025	0.786
Magnesium	-1.034	-0.020	0.826
Phosphorus	1.810	0.085	0.346
Gender	2.051	0.072	0.424
Group	3.838	0.220	0.036
Dependent variable: 25(OH)D			

## Discussion

This study aimed to assess vitamin D levels and some related biochemical parameters in pregnant women who smoke regularly during pregnancy, as well as in mothers and newborns who do not actively smoke but are exposed to second-hand smoke.

Diseases caused by malnutrition have come to the fore more in the last century [7]. Vitamin D deficiency is one of these diseases, and it has been one of the most researched nutrients [8]. It is well-documented that smoking during pregnancy may lead to vitamin D deficiency. A retrospective study conducted in Finland revealed that smoking during pregnancy caused a 27% decrease in serum 25(OH)D level from the first trimester [3]. In another study conducted in Iran, serum vitamin D levels of pregnant women and infants who were exposed to second-hand smoke and those who were not were evaluated. Although there was no significant difference in vitamin D levels between the groups, vitamin D levels were relatively lower in both the mother and the infant in the passive smoker group [2]. Likewise, in our study, vitamin D, calcium, and magnesium levels of mothers who smoked were significantly lower than those who were exposed to second-hand smoke and those who did not. Moreover, the vitamin D levels of mothers and babies exposed to second-hand smoke in the non-smoker group were significantly lower than mothers and babies who were not exposed to second-hand smoke. In the babies of these three groups, a significant decrease was observed only in vitamin D levels. This finding suggests that the factors involved in this change may be associated with maternal hemodilution rather than vitamin D [9].

Preeclampsia is one of the common causes of hypertension in pregnancy, and an important cause of preterm birth. There are various studies investigating the relationship between smoking during pregnancy and hypertension and preeclampsia. For example, England et al. (includes 48 epidemiological studies from 1959 to 2006) and Conde-Agudelo et al. showed a negative correlation between smoking during pregnancy and preeclampsia (including 35 studies from 1966 to 1998). In other words, it has been revealed that the risk of preeclampsia is lower in pregnant smokers [10]. Another review showed that smoking during pregnancy was a risk factor for hypertension in Asia, whereas it was a protective factor in Europe and North America [11]. In our study, when compared to the other two groups, the systolic and diastolic blood pressures and proteinuria levels were significantly higher in the mothers who gave birth earlier, despite being term. Another important cause of hypertension in pregnancy is vitamin D deficiency. In another prospective study we conducted before, we determined that vitamin D levels were lower in preeclamptic pregnant women [6]. According to an article published in 2021, the findings suggested that vitamin D deficiency and passive smoking during pregnancy might have a synergistic impact on gestational hypertension [12]. Thus, it should be kept in mind that vitamin D deficiency and smoking may be important interrelated factors for hypertension in pregnancy.

Birth weight, height, and head circumference are the main indicators used to evaluate fetal growth. It has been demonstrated that these parameters may be delayed due to the mother's smoking during pregnancy [13]. Besides, the APGAR score, which is also one of the frequently utilized indexes, is used to evaluate the general condition of the newborn in the first and fifth minutes after birth. There are various studies suggesting that smoking during pregnancy might be associated with a low APGAR score at birth [14]. In a study conducted in Jordan, it was stated that active or passive smoking during pregnancy might cause lower birth weight, height, head circumference, and APGAR score in infants [15]. Similarly, in our study, the weight, height, and one-minute APGAR scores of babies of smoking mothers were significantly lower than babies of mothers who were exposed to second-hand smoke and did not smoke. However, no significant difference was found between the groups regarding head circumference.

Although the prospective design of our study is its important strength, the relatively small number of participants, the absence of different ethnic origins, the limited number of samples, and possible selection bias are the limitations of this study. We also did not have the resources to measure biomarkers of the vitamin D endocrine system, parathyroid hormone concentration, or other functional indicators of maternal and newborn vitamin D status. Unmeasured confounders or errors in measuring existing confounding factors might bias our results.

## Conclusions

Vitamin D deficiency and exposure to second-hand smoke remain major public health problems. The co-occurrence of these two problems may cause severe health problems, especially in pregnant women and their infants. This study shows that exposure to cigarette smoke, whether active or second-hand, can lead to low vitamin D levels in both mother and infant. When determining the health program of the countries, it is of great importance to pay attention to the vitamin D supplement intake of pregnant women and infants and take measures that will cause less exposure of these groups to smoking.
